# Adaptation to random and systematic errors: Comparison of amputee and non-amputee control interfaces with varying levels of process noise

**DOI:** 10.1371/journal.pone.0170473

**Published:** 2017-03-16

**Authors:** Reva E. Johnson, Konrad P. Kording, Levi J. Hargrove, Jonathon W. Sensinger

**Affiliations:** 1 Department of Mechanical Engineering, Valparaiso University, Valparaiso, Indiana, United States of America; 2 Rehabilitation Institute of Chicago, Chicago, Illinois, United States of America; 3 Department of Physical Medicine and Rehabilitation, Northwestern University, Chicago, Illinois, United States of America; 4 Department of Physiology, Northwestern University, Chicago, Illinois, United States of America; 5 Institute of Biomedical Engineering, University of New Brunswick, Fredericton, New Brunswick, Canada; 6 Department of Electrical and Computer Engineering, University of New Brunswick, Fredericton, New Brunswick, Canada; Duke University, UNITED STATES

## Abstract

The objective of this study was to understand how people adapt to errors when using a myoelectric control interface. We compared adaptation across 1) non-amputee subjects using joint angle, joint torque, and myoelectric control interfaces, and 2) amputee subjects using myoelectric control interfaces with residual and intact limbs (five total control interface conditions). We measured trial-by-trial adaptation to self-generated errors and random perturbations during a virtual, single degree-of-freedom task with two levels of feedback uncertainty, and evaluated adaptation by fitting a hierarchical Kalman filter model. We have two main results. First, adaptation to random perturbations was similar across all control interfaces, whereas adaptation to self-generated errors differed. These patterns matched predictions of our model, which was fit to each control interface by changing the process noise parameter that represented system variability. Second, in amputee subjects, we found similar adaptation rates and error levels between residual and intact limbs. These results link prosthesis control to broader areas of motor learning and adaptation and provide a useful model of adaptation with myoelectric control. The model of adaptation will help us understand and solve prosthesis control challenges, such as providing additional sensory feedback.

## Introduction

Reducing movement errors is a fundamental goal of human learning, but is difficult for amputees using electromyographic (EMG) signals to control powered upper limb prostheses [1]. Errors may be either random (caused by unpredictable temporary changes), or systematic (caused by altered or incorrect estimation of task dynamics). To minimize overall error, random errors should be ignored, whereas systematic errors should result in adaptation of the movement [2,3]. Thus when an error occurs, the person needs to decide if, and how much to adapt the next movement. This decision may be especially difficult when using a myoelectric interface, which involves frequent errors, reduced sensory feedback, unfamiliar dynamics, and highly variable control signals. Adapting appropriately to errors is crucial for improving performance; thus, we need to study adaptation during prosthesis use in order to develop tools to help amputees reduce errors and complete tasks skillfully.

Adaptation studies have influenced the rehabilitation of patients with neurological disorders, and if applied correctly, promise insights into how prosthesis control systems can best communicate with the amputee’s neuromuscular system. Healthy, able-bodied persons continuously adapt during all types of movements, which enables them to flexibly reduce error in many changing scenarios [4–6]. This natural ability is disrupted in patients with neurological disorders, but adaptation studies have helped clarify how function is impaired [7] and how rehabilitation can best aid natural recovery processes [8–11]. For amputees, studying adaptation behavior will clarify the capabilities of the human and prosthesis as an integrated system and guide necessary improvements. Two significant knowledge gaps must be addressed: (1) whether adaptation is altered for a myoelectric control interface compared to able-bodied joint angle and torque control interfaces, and (2) whether adaptation is altered for an amputee’s residual limb compared to the intact limb. This paper addresses both questions by comparing adaptation behavior across myoelectric, joint angle and joint torque control interfaces in non-amputee and amputee subjects.

A central theory of motor learning is that the brain estimates properties of the body and its surroundings by considering uncertainty—a process described by Bayesian models [12,13]. The brain keeps track of relevant information, such as environmental characteristics (how heavy is the object I’m holding), feedforward predictions (based on my knowledge of my body’s dynamics, where should my hand be after a movement), and sensory feedback (where is my hand, according to my eyes and proprioception). Because of variability in these information sources, the brain can form better estimates by also keeping track of the uncertainty of each source [14]. Sources with more uncertainty are weighted less in the combined estimate of a property. For example, to estimate the hand’s distance from a target, the brain relies on sensory feedback information during an unfamiliar task when feedforward uncertainty is high and, conversely, relies on feedforward predictions when feedback uncertainty is high. Once an estimate of hand position and error is formed, the brain must decide if, and how much to adapt to an error.

Adaptation is a much studied process [15], and Bayesian modeling is one popular method of describing how adaptation is affected by uncertainty [12]. One common model, the Kalman filter, can be used to model a potential strategy of how to respond optimally to random and systematic errors [16,17]. In the Kalman framework, the brain should assign greater uncertainty to more variable information and processes, which are typically the cause of random errors [18,19]. By doing so, the system responds slowly to errors and avoids overcorrecting to random variability [20]. In this study we describe a similar but modified Kalman filter model (see Methods for details), and use this model to evaluate how efficiently subjects adapted to errors with each control interface.

Typical adaptation studies involve subjects performing reaching movements [5,21,22], but for amputees the natural motor system interfaces with artificial controllers and actuators. Non-amputee subjects move their arms by coordinating joint angles and joint torques, whereas amputees move powered prosthetic arms using a myoelectric control interface [23] (in which contraction of residual muscles in the amputated limb modulates electromyographic (EMG) signals, which in turn control prosthesis joint velocity [24,25]). Current myoelectric control systems are not always able to decode movement intent accurately, which, combined with variability in the EMG control signals, can result in unexpected prosthesis movements [26]. Although EMG signals are considered the best available control interface for powered prostheses [27], they introduce significant feedforward variability compared to joint angle and torque signals [28]. Thus, myoelectric prosthesis control causes more random errors of movement, which should increase uncertainty and may alter adaptation behavior.

EMG control may also provide less feedback information. Using joint angle control activates the body’s full set of proprioceptive feedback sensors, whereas isometric torque and EMG control do not (i.e., isometric contractions do not fully activate muscle spindles, which signal length and velocity changes [29]). When using joint torque control, users have direct feedback on joint torque through muscle force sensors (Golgi tendon organs); however, EMG is a byproduct of muscle contraction. EMG amplitude is roughly proportional to torque for isometric contractions [30], so muscle feedback loops should provide similar information for EMG and torque, but there may be enough difference to increase error and uncertainty. Thus angle, torque, and EMG control may give the user progressively less feedback information. To study how subjects process feedback uncertainty with each control interface, we manipulated the uncertainty of visual feedback and observed the effect on adaptation behavior.

In this paper we focused on how adaptation to random and systematic errors may differ when controlling a prosthesis compared to an intact limb, in order to address difficulties in reducing movement errors with a myoelectric interface. In the experimental protocol, we introduced random visual perturbations for two reasons: (1) to observe how subjects respond to random errors, and (2) to increase task difficulty and ensure that subjects continued to make systematic errors. Self-generated errors (errors made by the subjects themselves) include both systematic errors (e.g., from misestimating the effort needed to reach the target), and random errors (e.g., from neural signal variability). Thus, subjects needed to estimate how much to adapt to each perceived error, based on the uncertainty in their feedforward and feedback information.

We studied how this adaptation differed across control interfaces in non-amputee subjects using angle, torque, and EMG control signals, and in amputee subjects using EMG control with their intact and residual limbs. Adaptation was analyzed as a linear function of self-generated errors, perturbations, and visual feedback uncertainty. We previously studied adaptation in non-amputee subjects [31] and presented preliminary analyses with amputee subjects [32]. In this work, we compare adaptation across non-amputee and amputee subjects, and test a model that describes adaptation across all interfaces. The experimental results from each control interface were compared to the theoretical predictions of a hierarchical Kalman filter model that was designed to adapt efficiently to random and systematic errors. In short, this work enables relevant, principled comparisons between how non-amputee subjects adapt when controlling their own joint angles, and how amputee subjects adapt when using EMG control.

## Methods

### Subjects

Eight subjects with transhumeral amputations and eight non-amputee subjects participated in this experiment, which was approved by the Northwestern University Institutional Review Board. All subjects gave written consent for participation. Amputee subjects were all male and the average age was 43 years with a standard deviation of 16 years. The average age of non-amputee subjects (three female, five male) was 27 years with a standard deviation of 3 years. The data obtained from the non-amputee subjects was also analyzed in our previous work [31].

### Protocol

**Fig 1:**
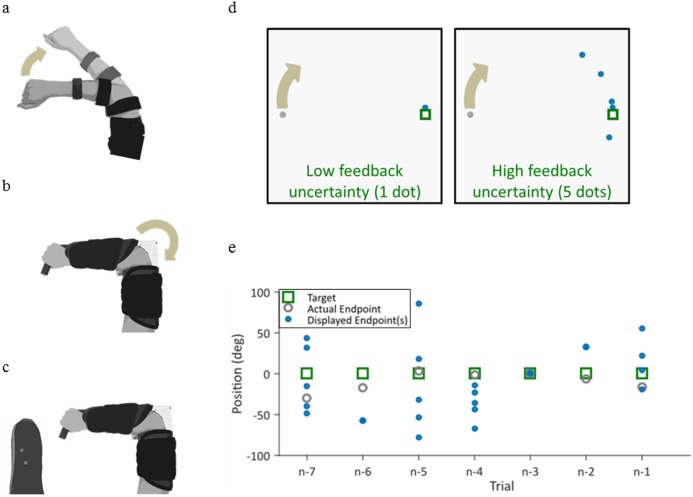
Experimental setup. Subjects used elbow extension to move a cursor with different control interfaces. Non-amputee subjects performed the protocol three times, once each using joint angle (a), joint torque (b), or EMG (c) to control the cursor. The angle control interface involved isotonic contractions, whereas the torque and EMG control interfaces both used isometric contractions. Amputee subjects performed the protocol once with the residual limb and once with the intact limb using EMG to control the cursor (c). The cursor moved along a 1DOF circular track (d). Feedback uncertainty was randomly manipulated by displaying the cursor as one dot (low uncertainty) or five dots (high uncertainty). Cursor position for an illustrative subject was perturbed visually by either -40, 0, or 40 degrees (e), where n is the most recent trial in the series.

The task was described previously with non-amputee subjects [31] and originally modified from [19]. Subjects sat comfortably in front of a computer display screen (Fig 1d). They used elbow extension movements to control a virtual cursor along a single degree-of-freedom (DOF) circular track (radius = 13 cm). The cursor started at the left side of the circle (180 degrees) and a target remained stationary at the right side of the circle (0 degrees). The start of each trial was indicated by an audio signal triggered by the experimenter. Subjects had 3 seconds to move the cursor from the starting position to the target. The cursor then returned to the starting position.

Each experiment comprised three phases: familiarization, training, and testing. The familiarization phase consisted of 10 trials, in which the cursor was displayed as one dot that was unperturbed and visible throughout the movement. In the training phase, the cursor was still unperturbed and displayed as one dot, but visual feedback was taken away 0.5 seconds into the movement time. The cursor reappeared after the trial to give 100 ms of terminal feedback (similar to [19,33] and others]). Training continued until the subject was able to complete 10 trials with an average error of under 20 degrees (this usually took 15–20 trials). In the testing phase, subjects were given only terminal visual feedback of the cursor, which was randomly visually perturbed with two levels of feedback uncertainty (described further in the following paragraphs). The testing phase included 4 blocks of 75 trials each, with approximately 2 minutes of rest between blocks.

During the testing phase (Fig 1e), visual perturbations were applied to the displayed cursor endpoint. Perturbations were randomly assigned to either -40, 0, or 40 degrees. Subjects were encouraged to hit the target as accurately as possible and were told that the visual feedback provided represented the true cursor position.

Two levels of feedback uncertainty were created by displaying the cursor as either one dot or five dots (Fig 1d), an approach used previously by [19,34–36]. When subjects saw a one-dot cursor, feedback uncertainty was low. When subjects saw five dots, feedback uncertainty was high. The location of the five dots was randomly selected from a Gaussian distribution with the mean as the cursor position and a standard deviation of 40 degrees. The level of feedback uncertainty was randomly assigned on each trial.

Several paradigms are commonly used to study adaptation [37]; here we chose to observe trial-by-trial adaptation to random visual perturbations. This approach allowed us to study how much the subject corrected to error on each individual movement [38]. Furthermore, we simplified the correction by displaying visual feedback only at the end of each movement, which minimized online feedback corrections, and allowed us to perturb movement without the subject’s knowledge [19,33,39]. The target remained stationary on each trial. Thus the subject’s response to the random perturbation, as well as to the error in their own movement (which we refer to as self-generated error), was primarily represented by the movement on the next trial.

### Control interfaces

Non-amputee subjects completed the protocol three times: once each using angle, torque, and EMG control signals, in a randomized order (Fig 1a and 1b). In the joint angle control interface, the subject extended the right elbow while an electrogoniometer (Biometrics Ltd) measured the elbow angle. The subject’s view of their arm was blocked. The angle output of the goniometer was filtered with a low-pass cutoff frequency of 50 Hz. Elbow flexion of 45° to 135° was mapped to 360° of the circular cursor track. In the torque and EMG control interfaces, the subject’s right arm was strapped into a modified elbow brace that restricted motion (ProCare Elbow RANGER Motion Control), and the subject generated isometric extension torque about the elbow. Elbow extension torque was measured by a reaction torque sensor (Futek TFF40). EMG activity of elbow extension was measured by a self-adhesive bipolar electrode (Delsys Bagnoli) placed over the lateral head of the triceps brachii.

Amputee subjects completed the protocol two times: once using EMG control with their intact limb and once using EMG control with their residual limb, in a randomized order (Fig 1c). For the intact limb, the experimental set-up was identical to that for non-amputee subjects using EMG control. For the residual limb, subjects donned a gel liner with embedded snap electrode contacts (Delsys, custom-made). The snap electrodes were placed over each subject’s myoelectric control site for elbow extension, as identified by a trained prosthetist. The effort level required to control the cursor was equalized as closely as possible between each subject’s residual and intact limbs, although this was difficult to achieve.

EMG control signals were normalized to the mean absolute value over 10 seconds of a medium strength contraction. Control signals were high-pass filtered at 0.1 Hz, rectified, low-pass filtered at 5 Hz, normalized, and mapped to cursor angle with the following transfer function:

$$\frac{\theta \left(s\right)}{u\left(s\right)}=\frac{1250}{s^{2}+11s}$$ (1)

These dynamics act as a common clinical EMG filter [40]; particular values were chosen to emulate velocity control [27] and the dynamics of a typical powered prosthetic arm, the LTI Boston Digital^™^ elbow [41].

### Analysis of adaptation

We analyzed the individual trial-by-trial corrections to both self-generated errors and perturbations. To do so, we ran a linear regression to determine the contributions of each factor to the self-generated error, using the following equation:

$$Error\left(n+1\right)-Error\left(n\right)=b_{0}+b_{1}Error\left(n\right)+b_{2}Perturb\left(n\right)+b_{3}Feedback\left(n\right)\times Error\left(n\right)\;+\;b_{4}Feedback\left(n\right)\times Perturb\left(n\right)$$ (2)

where n is the trial number, Error is a continuous variable representing self-generated error, Perturb is a discrete variable representing the visual perturbation of either -40°, 0°, or 40°, and Feedback is a discrete variable that equals 1 for high feedback uncertainty (five dots) trials, and 0 for low feedback uncertainty (one dot) trials.

### Modeling

We used a hierarchical structure of two Kalman filters to describe adaptation: one for state estimation described by equations (3–9) in Table 1 and one for parameter estimation described by equations (10–14) in Table 2. The state estimation model describes the subject’s estimate of cursor position at the end of each trial, based on both the predicted and observed state. The predicted state is made possible by the parameter estimation model, which describes how the subject updates an estimate of relevant parameters—in this case, the relevant parameter is the effort needed to reach the target. This parameter represents what is often called the forward or internal model [42,43].

**Table 1:** State estimation model.

Predict state and covariance	
${\hat{x}}_{n}^{\prime }=\hat{A}x_{n-1}+\hat{B}u$	(3)
$P_{n}^{\prime }=\hat{A}P_{n-1}{\hat{A}}^{T}+Q$	(4)
Perform movement	
$\begin{array}{cc} x_{n}=Ax_{n-1}+Bu+\varepsilon , & p\left(\varepsilon \right)\sim N\left(0,Q\right) \end{array}$	(5)
Observe movement	
$\begin{array}{cc} z_{n}=Hx+\nu , & p\left(\nu \right)\sim N\left(0,R\right) \end{array}$	(6)
Correct state prediction and covariance	
$K_{n}=P_{n}^{\prime }H^{T}{\left(HP_{n}^{\prime }H^{T}+R\right)}^{-1}$	(7)
${\hat{x}}_{n}={\hat{x}}_{n}^{\prime }+K_{n}\left(z_{n}-H{\hat{x}}_{n}^{\prime }\right)$	(8)
$P_{n}=P_{n}^{\prime }\left(I-K_{n}H\right)$	(9)
**State Estimation Variables**	
*x*: state	
$\hat{x}$: estimated state using estimated parameters (′ indicates prediction or prior)	
*A*, *B*: system dynamics	
$\hat{A},\;\hat{B}$: estimated dynamics, composed of estimated parameters: $\;\hat{A},\;\hat{B}=f\left(params\right)$	
*u*: control signal	
*P*: state estimate uncertainty	
*Q*: process noise	
*z*: sensory feedback information	
*H*: observation matrix	
*R*: measurement noise	
*K*: Kalman gain	

**Table 2:** Parameter estimation model.

Factor in forgetting and uncertainty from trial to trial	
$params=A_{param}params$	(10)
$P_{param}=A_{param}P_{params}A_{param}{}^{T}+Q_{param}$	(11)
Update parameters and uncertainty	
$K_{param}=\frac{P_{param}H_{param}{}^{T}}{\left(H_{param}P_{param}H_{param}{}^{T}+R_{param}\right)}$	(12)
$params=params+K_{param}\left(\hat{x}-{\hat{x}}^{\prime }\right)$	(13)
$P_{param}=P_{param}(I-K_{param}H_{param})$	(14)
**Parameter Estimation Variables**	
*params*: parameters	
*P*_*param*_: propagated uncertainty of parameters	
*Q*_*param*_: uncertainty of parameters	
*R*_*param*_: Uncertainty of sensory information used to update parameters: *R*_*param*_ = *Q* + *R*	
*K*_*param*_: Kalman gain	
*H*_*param*_: mapping of parameters to states	
*A*_*param*_: forgetting factor	

For the state estimation model (Table 1), we used the standard Kalman algorithm [16,17]. We calculated the actual state (where the cursor stopped) using the programmed dynamics parameters of the virtual environment. We also calculated the estimated state (where the subject thinks the cursor stopped, before getting feedback) by modeling the dynamics parameters the subjects estimated (described in the parameter estimation model). The sensory feedback is a fused maximum likelihood estimate of visual feedback (includes perturbations) and proprioceptive feedback (does not include perturbations) [44].

The parameter estimation model (Table 2) was based on the Kalman algorithm and modified originally from [18]. We changed the parameter update equation (equation 13) to adapt parameters based on the difference between the corrected state $\hat{x}$ and the predicted state ${\hat{x}}^{\text{'}}$ (instead of the difference between the observed state *z* and the predicted state ${\hat{x}}^{\text{'}}$). This reflects the belief that people integrate sensory feedback with their feedforward prediction, and use this corrected state as their best estimate of any error [19,42].

We also introduced a definition for *R*_*param*_ in the parameter update equations. *R*_*param*_ represents the subject’s uncertainty in the sensory information used to update the parameters. We set *R*_*param*_ equal to the sum of the process noise *Q* and the measurement noise *R*. This relationship reflects the belief that subjects should rely heavily on sensory feedback information to update their internal model when they estimate low system variability (*Q*) and low feedback uncertainty (*R*) [2]. However, when either system variability or feedback uncertainty is high, *R*_*param*_ increases, which decreases *K*_*param*_, and thus subjects should adapt more slowly to any observed difference between the corrected and predicted state.

The hierarchical Kalman model was implemented in a simple 1-DOF form for trial-by-trial adaptation to random visual perturbations (with cursor position as the state variable). The model was fit to data observed from non-amputee subjects using joint angle-based control. From there, only the process noise parameter Q in the state estimation model was changed to fit the data from the other control interfaces. Our code and parameter values are available online from the Dryad Digital Repository: http://dx.doi.org/10.5061/dryad.b2p3j and in the Supporting Information section (S1 File).

## Results

We first report the modeling findings to give a broad sense of how different factors are predicted to influence adaptation. We then report our experimental observations of how our subjects adapted to self-generated errors, perturbations, and feedback uncertainty, with modeling comparisons for each.

### Modeling

We used modeling to investigate the factors that influence adaptation, focusing on self-generated errors, perturbations, and feedback uncertainty. To construct a model, we considered the fact that subjects needed to estimate both the position of their cursor (which can be modeled by a Kalman filter [42]) and the properties of their sensorimotor system (which can also be modeled by a Kalman filter [2,18,19]). We thus constructed a hierarchical Kalman filter and implemented it for a simple 1-DOF trial-by-trial adaptation to errors and random perturbations.

**Fig 2:**
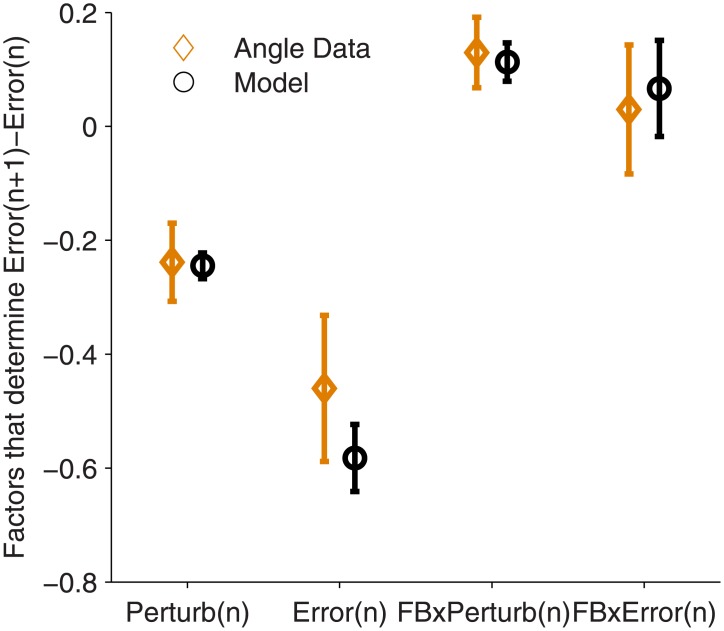
Hierarchical Kalman model describes adaptation behavior of subjects using joint angle-based control. The model was used to predict behavior on the protocol described in the methods (Tables 1 and 2), and a linear regression was used to analyze the factors that determined adaptation on each trial: Error(n+1)-Error(n) = b_0_ + b_1_Error(n) + b_2_Perturb(n) +b_3_Feedback(n) x Error(n) + b_4_Feedback(n) x Perturb(n) (Eq 2). This plot shows the linear regression coefficients of the model predictions (+/- standard deviation) compared to the observed linear regression coefficients of subjects using the joint angle control interface (+/- standard error of the mean).

We fit our model to data from non-amputee subjects using joint angle to control the prosthesis, and found that it predicted the observed adaptation to self-generated errors and perturbations (Fig 2). The model shows that subjects should adapt more to self-generated errors than to perturbations (Fig 2, Perturb(n) and Error(n) factors). Intuitively, one should adapt to systematic errors and completely ignore random errors. However, visual feedback displays the sum of the systematic self-generated errors and the perturbations, which complicates the task of deciding how to respond. Thus our subjects, and the model, had to estimate the best adaptation response with the available information.

When visual feedback uncertainty is increased by showing a cloud of 5 dots instead of 1, the model shows that subjects should adapt less to errors and perturbations (Fig 2, FBxPerturb(n) and FBxError(n) factors). In modeling terms, larger R decreases both K and *K*_*param*_. Overall, the responses of the Kalman filter model matched how our subjects responded, suggesting that our subjects made efficient decisions when using joint angle-based control.

**Fig 3:**
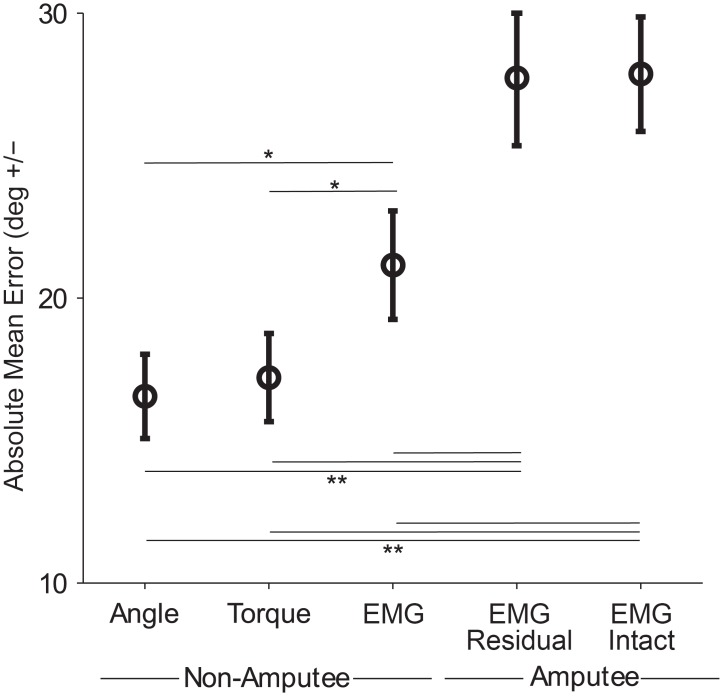
Absolute mean error of non-amputee subjects using angle, torque, and EMG control and amputee subjects using EMG control with the residual and intact limbs. Mean error refers to the unperturbed distance between cursor and target at movement endpoint, averaged over all trials for each subject using each control interface. Bars show standard error of the mean. * indicates p<0.05, one-way ANOVA with Tukey post-hoc tests. ** indicates p<0.05, unpaired t-tests.

**Fig 4:**
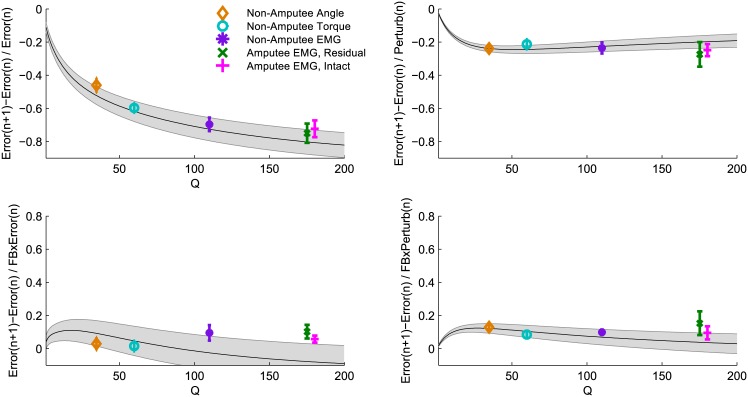
Hierarchical Kalman model predicts adaptation behavior across control interfaces by changing only the process noise parameter Q of state estimation. For each value of Q ([1:200]), the model was run on 300 trials with random visual perturbations and randomly selected levels of low and high feedback uncertainty (cursor shown as 1 dot or 5 dots). A linear regression analysis was used for both the model data and the observed experimental data (Eq 2). Shaded curves show the linear regression coefficients (+/- standard deviation) of the model predictions. Data points show the linear regression coefficients (+/- standard error of the mean) of observed experimental data for each control interface. (a) shows adaptation to self-generated errors, which corresponds to coefficient b1 in Table 3. (b) shows adaptation to perturbations, which corresponds to coefficient b2 in Table 3. (c) shows the change in adaptation to self-generated errors due to high feedback uncertainty, which corresponds to coefficient b3 in Table 3. (d) shows the change in adaptation to perturbations due to high feedback uncertainty, which corresponds to coefficient b4 in Table 3.

A useful model should be applicable across a wide range of situations, so we compared the predictions of the model with experimentally observed adaptation across all control interfaces. To avoid overfitting the model to each control interface, we changed only one parameter (we discuss potential effects of changing other parameters in the feedback uncertainty section and in the Discussion). The control interface influenced error sizes that subjects made during the task (Fig 3), and thus we needed to adjust our model to account for these differences. To do so, we tuned the process noise *Q* of the state estimation model to the error levels of each control interface. We found that by changing just the process noise parameter, the model was able to accurately predict adaptation behavior across all control interfaces (Fig 4, all subplots).

Changing the process noise *Q* influenced adaptation to self-generated errors, perturbations, and feedback uncertainty in different ways. Increasing the process noise increased adaptation to self-generated errors (Fig 4a), but had little effect on adaptation to perturbations (Fig 4b). The effect of feedback uncertainty was minimally influenced by process noise, although an increase in process noise slightly decreased the effect of feedback uncertainty on adaptation to self-generated errors (Fig 4c and 4d). Other than the effect of feedback uncertainty on adaptation to self-generated errors (discussed later), these trends were reflected in both the model predictions and the observed data.

### Adaptation to systematic self-generated errors

**Fig 5:**
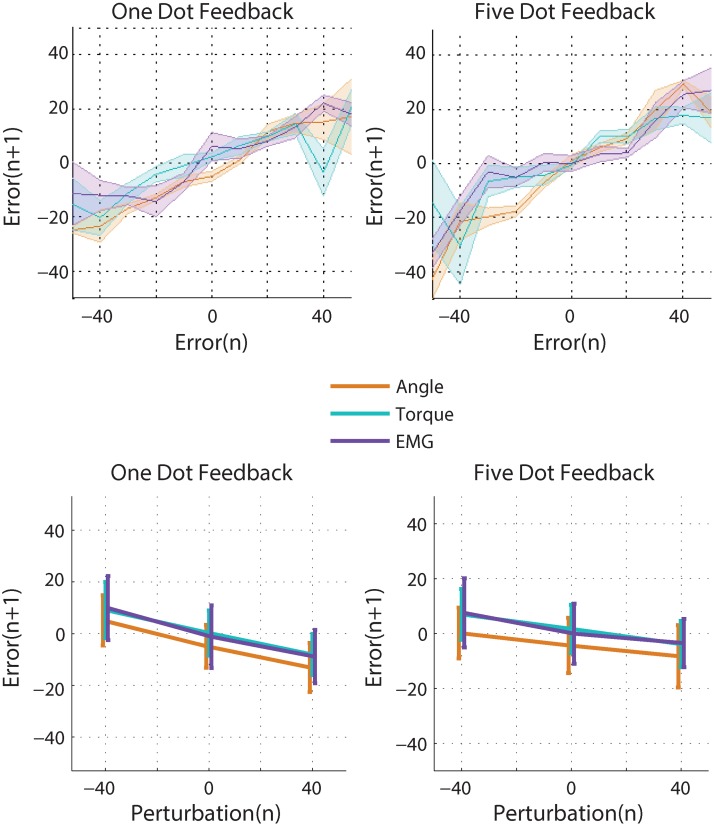
Non-amputee subjects’ responses to self-generated errors (top) and perturbations (bottom).

Efficient adaptation to self-generated errors is important for improving performance. In response to a self-generated error, subjects typically made a smaller error in the same direction on the following trial (Fig 5, top). They reduced the error by adapting their movement from one trial to the next, and this adaptation scaled linearly according to error size. Thus subjects corrected for a portion of self-generated error on each trial. Non-amputee subjects adapted to 46% of an error when using angle control, 56% when using torque control, and 70% when using EMG control (b_1_ regression term, each pairwise comparison p<0.05, repeated measures ANOVA, Table 3). In short, non-amputee subjects increasingly adapted across joint angle, joint torque, and EMG control interfaces, and this pattern matched that predicted by increasing the process noise estimate of our model (Fig 4a).

**Table 3:** Linear regression analysis of adaptation across control interfaces.

	Self-Generated Error		Perturbation		Feedback Uncertainty x Self-Generated Error		Feedback Uncertainty x Perturbation		
	b_1_	SEM	b_2_	SEM	b_3_	SEM	b_4_	SEM	r^2^
Angle	-0.460	0.016	-0.239	0.009	**	**	0.130	0.008	0.373
Torque	-0.598	0.010	-0.213	0.011	**	**	0.085	0.008	0.369
EMG	-0.696	0.014	-0.235	0.012	0.096	0.017	0.099	0.006	0.384
TH Residual	-0.750	0.021	-0.274	0.026	0.103	0.015	0.153	0.025	0.401
TH Intact	-0.723	0.018	-0.248	0.013	0.058	0.025	0.096	0.014	0.384

A linear regression was used to analyze how errors, perturbations, and feedback uncertainty on trial (n) determined the adaptation for trial (n+1). The following equation was fit to each subject: Error(n+1)-Error(n) = b_0_ + b_1_Error(n) + b_2_Perturb(n) +b_3_Feedback(n) x Error(n) + b_4_Feedback(n) x Perturb(n), where n is the trial number, Error is a continuous variable representing self-generated error, Perturb is a discrete variable representing the visual perturbation of either -40°, 0°, or 40°, and Feedback is a discrete variable that equals 1 for high feedback uncertainty (five dots) trials, and 0 for low feedback uncertainty (one dot) trials. This table shows the across-subject averages and standard errors of each coefficient, as well as the average r^2^ value for each control interface.

** indicates that these coefficients were insignificant for a majority of subjects. The intercept coefficient b_0_ was also found to be insignificant. (Note that here significance refers to the significance of the regression coefficients (i.e. how well they predict the regression output). For significant differences in regression coefficients, see Fig 7.

**Fig 6:**
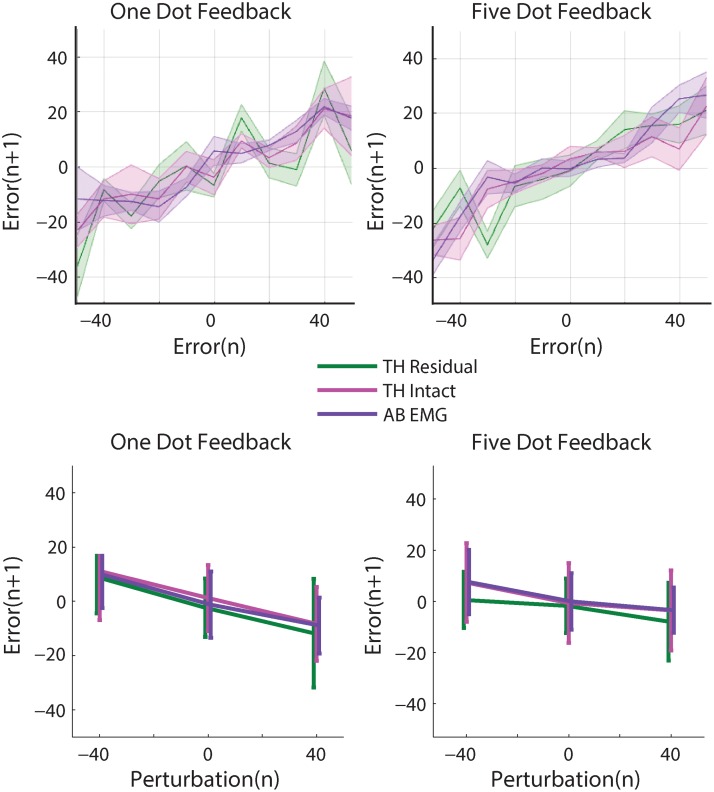
Amputee and non-amputee subjects’ responses to self-generated errors (top) and perturbations (bottom).

**Fig 7:**
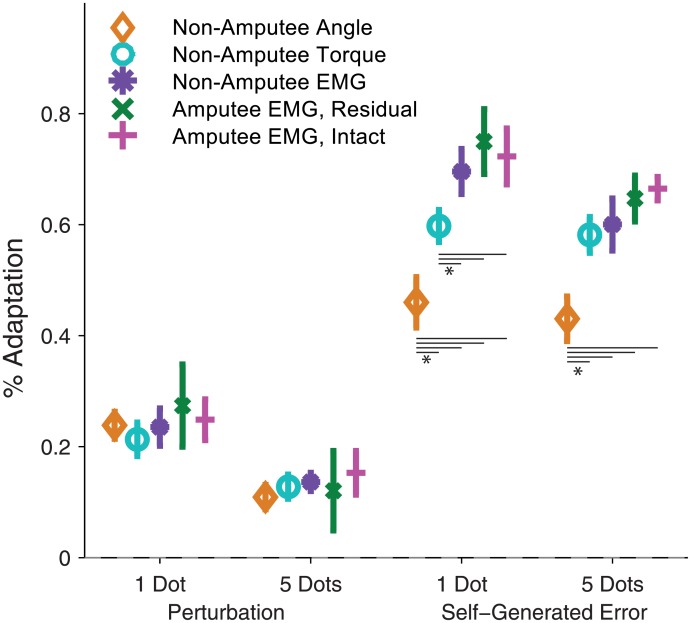
Adaptation to random perturbations and self-generated errors. This plot shows the magnitudes of the adaptation coefficients in Table 3. Because the feedback coefficients (b_3_ and b_4_) describe a change to adaptation, we summed them with the error and perturbation coefficients (b_2_ and b_3_) to display adaptation in response to high feedback uncertainty trials (5 dots). * indicates significant difference of p < 0, using repeated measures ANOVA.

How did an amputation affect adaptation to self-generated errors? Amputee subjects followed the same patterns of reducing a portion of self-generated error on each trial (Fig 6, top). This portion was 75% when using both the residual limb and the intact limb, which was not significantly different from non-amputee subjects using EMG control (b_1_ regression term, Table 3, Fig 7). In sum, adaptation to self-generated errors when using EMG control was similar across all EMG control interfaces: amputee subjects using the residual limb, amputee subjects using the intact limb, and non-amputee subjects.

### Adaptation to random perturbations

Adaptation to random perturbations gives us insight into how subjects are estimating systematic and random errors, and how this estimation is affected by control interface. When subjects were perturbed in one direction, they adapted to the perturbation and erred in the opposite direction on the next trial (Fig 5, bottom; Fig 6, bottom). The random perturbations were of a constant magnitude (40 degrees), and subjects corrected for a portion of each perturbation. Non-amputee subjects adapted to 24% of a perturbation using angle control, 21% using torque control, and 24% using EMG control (b_2_ regression term, no significant difference, Table 3, Fig 7). Amputee subjects adapted to 27% of a perturbation using the residual limb, and 25% using the intact limb (b_2_ regression term, no significant difference, Table 3, Fig 7). Thus the control interface had no influence on adaptation to random perturbations and amputees behaved similarly to intact-limbed subjects, which matches the predictions of our model (Fig 4b).

### Feedback uncertainty

Because of potential differences in feedback information with each control interface, we studied the role of feedback uncertainty. For our subjects, feedback uncertainty reduced adaptation to self-generated errors by 3.0% for angle control, 1.6% for torque control, 9.6% for EMG control, 10.3% for amputees using their residual limb, and 5.8% for amputees using their intact limb (b_3_ regression term, non-amputee subjects using EMG and amputees using their residual limb were significantly different from the other groups, Table 3, Fig 7). Feedback uncertainty reduced adaptation to perturbations by approximately 11% for all control interfaces (b_4_ regression term, no significance difference). Overall, we observed that feedback uncertainty reduced adaptation to self-generated errors and perturbations, with a greater effect on adaptation to perturbations. These patterns were largely similar to our model predictions (Fig 4c and 4d).

**Fig 8:**
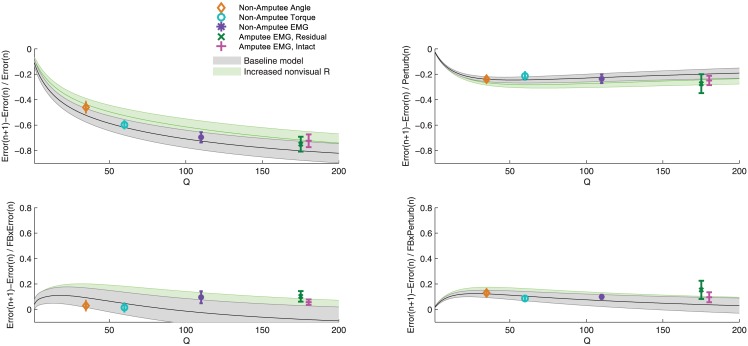
Predictions of hierarchical Kalman filter model for increased uncertainty of nonvisual feedback in state estimation. This figure is identical to Fig 4, but adds the patterns predicted if the feedback uncertainty of nonvisual feedback was increased.

However, one effect of feedback uncertainty does not match the patterns predicted by increasing the process noise estimate of our model. The model shows that as process noise increases, the effect of feedback uncertainty on adaptation to perturbations should decrease to near zero (Fig 4d). Instead, for our experimental data, the effect of feedback uncertainty increased for all three EMG control interfaces. We reasoned that the major difference between EMG and torque control interfaces (other than variability, which is modeled by process noise) is that EMG control involves less nonvisual feedback information. We tested this hypothesis by increasing the feedback variance *R* of the proprioceptive feedback in the state estimation model. We found that this change explained a number of trends: higher errors, decreased adaptation to self-generated errors, increased adaptation to perturbations, and increased effect of feedback uncertainty, which resulted in a better fit for all three EMG control interfaces (Fig 8). Thus, increasing process noise does not account for all of the patterns we observed, but increasing the estimate of nonvisual feedback variance *R* for EMG control explains the discrepancies.

## Discussion

Prosthesis control involves frequent movement errors, and amputees using powered prostheses often have difficulty reducing these errors. To better understand this difficulty and improve their ability to control their prosthesis, we studied adaptation—the process of adjusting behavior in response to errors. We focused in particular on how subjects distinguish between random errors and systematic errors, because this distinction may be complicated by the high variability of control signals and reduced feedback associated with prosthesis control. We observed how subjects adapted to self-generated errors and random visual perturbations with varying levels of feedback uncertainty. This paradigm, along with theoretical modeling of adaptation, allowed us to separate the effects of feedforward and feedback uncertainty.

We constructed a simple hierarchical Kalman model to describe efficient adaptation to both self-generated errors and random perturbations. Importantly, our model included separate processes for state estimation and for parameter estimation. This allowed us to distinguish between subjects’ estimate of system variability (process noise *Q*) and subjects' estimate of internal model uncertainty (parameter uncertainty *Q*_*param*_). In other words, this enabled modeling of a system where subjects are confident in their internal model parameters, but well aware that their control signals are noisy—and vice versa. Our model described adaptation patterns across control interfaces only by changing the process noise *Q* to account for differences in system variability (Fig 4).

Other models also tackle the problem of how to distinguish between random and systematic errors [18,45,46]. Our approach is especially relevant for prosthesis control, because it easily considers the effects of multiple sources of feedback information with varying levels of uncertainty. Thus our model can predict how different types of sensory feedback (and their perceived resolution) will influence both estimation and adaptation. Additional sensory feedback is a much-requested improvement for powered prosthesis control [47], and a principled understanding of the effects of feedback on error reduction will guide research and design efforts.

Adaptation to self-generated error increased across angle, torque, and EMG control interfaces (Table 3, Fig 7), which matches the patterns predicted by increasing the process noise parameter in the hierarchical Kalman model (Fig 4a). Thus, with EMG control, subjects are correctly estimating that their control involves more errors (Fig 3) (i.e., larger process noise), and thus that they need to rely more on visual and proprioceptive feedback to estimate their cursor position. As process noise increases, the difference between predicted and corrected position increases, and subjects rely more on this correction (larger Q increases K). The patterns we observed from increasing process noise agree with other studies that show humans are able to maintain accurate estimates of system variability and adjust adaptation accordingly [3,33,48,49]. This study is, to our knowledge, the first to show that this ability extends consistently across different control interfaces.

Adaptation to perturbations was not significantly influenced by control interface (Table 3, Fig 7), which again matches the patterns predicted by increasing the process noise parameter in the hierarchical Kalman model (Fig 4b). Note that for a typical Kalman filter model, increasing process noise Q should usually increase adaptation to errors—both self-generated and perturbations. However, because we defined R_*param*_ as the sum of process noise Q and feedback uncertainty R, increasing Q propagates through R_*param*_ to decrease K_*param*_, which reduces adaptation. This effect balances the increase in K and prevents over-adaptation to random errors caused by large process noise. If subjects *had* increased adaptation to perturbations, this would have indicated increased uncertainty of internal model parameters (i.e., increased *Q*_*param*_). In this task, with steady state dynamics, increasing *Q*_*param*_ would have caused increased adaptation to random perturbations, which only introduces further misestimation of the effort needed to reach the target. Thus the unchanging adaptation to perturbations indicates that subjects responded to perturbations efficiently. We found that adaptation behavior using EMG control was similar across non-amputee subjects and transhumeral amputees using both the residual and intact limbs (Table 3, Fig 7), but that amputee subjects performed the task with larger errors (Fig 3). This result was discussed previously [32] and may be consistent with our modeling predictions, which show a flattening of the self-generated error adaptation curve at higher error levels (larger Q values) (Fig 4a). However, we also have three alternative hypotheses. First, amputees may have higher system variability (larger Q, potentially due to higher average age of subjects [50,51]) and yet estimate internal model parameters with lower uncertainty (smaller *Q*_*param*_). In other words, amputees may have higher variability and yet be more confident in their control because they use EMG control every day. Second, half of the amputee subjects used their nondominant arm with the residual limb interface, and half used their nondominant arm with the intact limb interface, whereas all non-amputee subjects used their dominant arms. Use of the nondominant limb could have introduced more variability. Third, adaptation may not scale linearly with error size, instead, subjects may disproportionally respond to larger errors [52,53]. More rigorous modeling work, along with more complex tasks, could be used to further investigate these hypotheses.

The effect of feedback uncertainty was consistent across control interfaces for adaptation to perturbations, but was larger in EMG control for adaptation to self-generated errors (Table 3, Fig 7). The consistent response to perturbations is intuitive: since proprioceptive feedback loops cannot provide information on random perturbations, subjects relied on visual feedback to adapt to perturbations with all three control interfaces. However, the difference in adaptation to self-generated errors across control interfaces suggests differing feedback information. We hypothesized that with EMG control, subjects relied almost entirely on visual feedback, whereas with angle and torque control, proprioceptive feedback loops provided useful information. Our modeling results confirm this hypothesis, showing that the differences can be explained by increasing the estimate of nonvisual feedback uncertainty for EMG control (Fig 8).

If providing additional feedback compensates for the feedback uncertainty of EMG, our model predicts that subjects should be able to further reduce errors and improve their ability to distinguish between random and systematic errors. Note that in our experimental paradigm, self-generated errors include both systematic errors of misestimation and random errors due to imperfect decoding of EMG signals. Thus, what appears to be efficient behavior as predicted by the Kalman model may be over-adaptation to self-generated errors, if there are very high levels of random self-generated errors. One unique solution to combating this difficulty is to provide feedback on EMG signals themselves to aid subjects in determining the sources of errors with EMG control [54].

The task that subjects completed in this experiment required a simple single DOF movement and only one channel of EMG control signals. Thus this is a very basic, albeit necessary, first characterization of adaptation to random and systematic errors during prosthesis control. Future work should study multi-DOF movements and applied tasks to reveal more detailed adaptation behaviors, which may indicate that subjects do not always perform efficiently with EMG control. For example, with complex movements such as grasping, subjects seem to have more uncertain internal models with EMG compared to torque [55], thus in these cases additional feedback information becomes even more important [56].

In conclusion, this study showed that EMG control resulted in increased adaptation to self-generated errors, a behavior that was described by our hierarchical Kalman model by simply increasing the estimate of system variability. This agreement between experiment and model showed that subjects behaved efficiently with the available information. However, with more precise sensory feedback information, subjects should be able to further reduce errors and improve performance. These results link the challenges of prosthesis control to the broader literature of motor learning and adaptation, and provide a useful modeling tool for testing the effects of sensory feedback on powered prosthesis control.

## Supporting information

S1 File Modeling code and parameters. (PDF)
