# Supplementary material for: Adaptation to random and systematic errors: Comparison of amputee and non-amputee control interfaces with varying levels of process noise
Source: PLoS One. 2017 Mar 16;12(3):e0170473. doi: 10.1371/journal.pone.0170473 (PMC5354256; doi:10.1371/journal.pone.0170473)
Supplement: S1 File — (PDF) [file pone.0170473.s001.pdf]

Hierarchical Kalman Learning Model

The task is a 1-DoF movement to a target. You need to exert the right amount of effort to reach the target.

Here we use two layers of Kalman filters to describe learning during this task: state estimation and parameter estimation. The *state estimation* model uses the standard Kalman algorithm to describe where you land after a movement, based on both your predicted and observed state. The predicted state is made possible by the *parameter estimation* model. You maintain an estimate of the effort needed to reach the target, and this parameter is updated based on observed error. This parameter is often called the forward or internal model.

State Estimation

Mostly the standard Kalman algorithm (Kalman, 1960; Welch & Bishop, 2006). *Since we know the actual dynamics of the task, we keep track of the actual state (where the cursor lands) and the estimated state (where you think you land). The sensory feedback is a fused maximum likelihood estimate of visual feedback (includes perturbations) and proprioceptive feedback (does not include perturbations).*

State Estimation Variables	
x	state
$\hat{x}$	estimated state using estimated parameters ($'$ indicates prediction or prior)
A, B	system dynamics
$\hat{A}, \hat{B}$	estimated system dynamics, composed of estimated parameters: $\hat{A}, \hat{B} = f(params)$
u	control signal
P	state estimate uncertainty
Q	process noise uncertainty
z	sensory feedback information
H	observation matrix
R	measurement noise
K	Kalman gain
	shading indicates free parameters

Predict state and covariance

$$\hat{x}'_n = \hat{A}x_{n-1} + \hat{B}u$$

$$P'_n = \hat{A}P_{n-1}\hat{A}^T + Q$$

Perform movement

$$x_n = Ax_{n-1} + Bu + \varepsilon, \qquad p(\varepsilon) \sim N(0, Q)$$

Observe movement

$$z_n = Hx + \nu, \qquad p(\nu) \sim N(0, R)$$

Correct state prediction and covariance

Corrected by difference between observed & predicted feedback, weighted by K

$$K_n = P'_n H^T (HP'_n H^T + R)^{-1}$$

$$\hat{x}_n = \hat{x}'_n + K_n(z_n - H\hat{x}'_n)$$

$$P_n = P'_n(I - K_n H)$$

Parameter Estimation

Based on the Kalman algorithm. Modified from (Berniker & Kording, 2008) *with ideas from* (Wei & Kording, 2010) *and* (Burge, Ernst, & Banks, 2008) *(see Methods section for more information).*

Paramer Estimation Variables	
$params$	parameters that describe relevant dynamics
P_{param}	uncertainty of parameters
Q_{param}	uncertainty of parameters
R_{param}	Uncertainty of sensory information used to update parameters. $R_{param} = Q + R$
K_{param}	Kalman gain that weights how much you u
H_{param}	mapping of parameters to states
A_{param}	forgetting factor, or how each parameter varies from trial to trial
	shading indicates free parameters

Factor in forgetting and uncertainty from trial to trial

$$params = A_{param}params$$

$$P_{param} = A_{param}P_{param}A_{param}{}^T + Q_{param}$$

Update parameters and uncertainty

Updated by weighted difference between corrected and predicted state

$$K_{param} = P_{param}H_{param}{}^T / \left(H_{param}P_{param}H_{param}{}^T + R_{param}\right)$$

$$params = params + K_{param}(\hat{x} - \hat{x}')$$

$$P_{param} = P_{param}\left(I - K_{param}H_{param}\right)$$

Simple 1-DoF Implementation for Trial-by-Trial Visual Perturbations

This is a simple 1DOF implementation of the hierarchical Kalman filter model. For our experiment, we only analyzed the endpoints of movements, so we did the same for our modeling. However, this model can be expanded to describe movement trajectories by including system dynamics in the A and B matrices (and thus also the params vector).

Inputs:

Task Parameters

$target = 180 \text{ deg}$

$perturbations = [-40, 0, 40] \text{ deg}$

$n = 300 \text{ trials}$

State Estimation

$x = cursor\ position$

$A = 0,\ B = target,\ H = 1$

$P' = 0$ at the start of each movement

$\hat{B}(i) = param(i)$

$u(i) = target/\hat{B}(i)$

$z(i) = MLE\{z_{visual}(i), z_{proprioceptive}(i)\}$

Parameter Estimation

$A_{param} = H_{param} = 1$

$param(1) = target$ (subjects had practice trials with no perturbations to learn the task)

Uncertainty Parameters:

$R_{visual} = 5^2 \text{deg}^2$ or 8^2 deg^2 (trials were randomly assigned as low or high visual feedback uncertainty)

$R_{position} = 10^2 \text{deg}^2$ (estimate of feedback uncertainty with proprioception)

$Q_{param} = 19 \text{ deg}^2$

Q was set for each control signal based on the average mean absolute error of the task with no perturbations (values chosen from experimental mean absolute error of each control interface during training, before perturbations were introduced)

$Q\ (\text{deg}^2)$

Non-amputee subjects			Amputee Subjects	
Angle	Torque	EMG	Residual Limb	Intact Limb
35	60	110	175	180

Algorithm as implemented in MATLAB:

```
for i=1:n
    %State Estimation
    u(i) = targ/Bhat;                                   %control signal you think will hit target
    xhat_(i) = Bhat*u(i);                               %predicted movement
    P_ = Q;                                             %predicted variance

    x(i) = B*u(i) + sqrt(Q)*randn;                      %actual movement
    err(i) = x(i) - targ;                               %actual mvmt error

    zvis(i) = x(i) + pert(i) + sqrt(Rvis(i))*randn;     %visual feedback includes perturbation
    zpos(i) = x(i) + sqrt(Rpos)*randn;                  %proprioception observation
    Wvis = Rpos / (Rvis(i)+Rpos);                       %visual feedback weight
    Wpos = Rvis(i) / (Rvis(i)+Rpos);                    %proprioception weight
    z(i) = Wvis*zvis(i) + Wpos*zpos(i);                 %fused observation
    R = Rvis(i)*Rpos/(Rvis(i)+Rpos);                    %fused feedback uncertainty

    K = P_/(P_+R);                                      %calculate Kalman gain
    xhat(i) = xhat_(i) + K*(z(i)-xhat_(i));             %correct state estimate

    %Parameter Estimation
    Rparam = Q+R;
    Pparam = Pparam + Qparam;
    Kparam = Pparam/(Pparam+Rparam);
    param = param + Kparam*(xhat(i)-xhat_(i));          %Update parameter
    Pparam = Pparam*(1-Kparam);                         %Update parameter uncertainty
    Bhat = param;                                       %Transfer updated parameter to state estimator
end
```
